# The Hampstead General Hospital

**Published:** 1906-03-24

**Authors:** F. R. Humphreys

**Affiliations:** Chairman, Hampstead Division, British Medical Association


### Editor's Letter-Box.

Our Correspondents are reminded that prolixity is a great bar to publication, and that brevity of style and conciseness of statement greatly facilitate early insertion.]

### THE HAMPSTEAD GENERAL HOSPITAL.

*To the Editor of* The Hospital.

Sir,—The management of the Hampstead General Hospital has been the subject of much criticism by the local medical profession for some time past, and the matter has been coming to a head gradually in the course of the past year or two.

The hospital was originally founded by a local general practioner as a Home Hospital, where individual local practitioners could themselves continue, under more satisfactory circumstances, the treatment commenced in the patients' homes. The hospital was in considerable difficulties for some years; finally it was only by the united efforts of the local profession that it was kept open.

From a Home Hospital this institution has advanced, or degenerated, into a "General Hospital," with a large out-patient department, limited staff of general practitioners, a few (pay) beds for the patients of practitioners not on the staff, and a management on which the outside profession is unrepresented. This divorce between the management and the local profession is greatly to be deprecated on every ground.

The hospital has recently become possessed of a very large donation, and it is rumoured that it is about to receive another. It has been transferred to a new specially-erected building quite recently. The lines upon which the hospital authorities have been recently developing that institution indicate further alterations in the near future, and, indeed, there are rumours, apparently well-founded, which foreshadow further drastic changes.

This letter is not for the purpose of opening a discussion in your columns, Sir, but in order to request the general practitioners of Hampstead and its neighbourhood to keep an open mind upon the subject, in view of the proposal to call a meeting of the local profession in the course of the next few weeks to consider the whole matter.

I will, therefore, merely observe that there appears to be a concensus of medical opinion that an out-patient department in this neighbourhood is neither necessary nor desirable; that if any hospital be required in Hampstead it should be one of the nature of a Home Hospital; that the appointment of a small privileged staff is unfair to the patients of other practitioners, as indeed it is to the practitioners themselves (though the staff by all reports do their work in a very satisfactory manner); and that the appointment of a staff of consultants, which is rumoured as a possibility, in place of the existing general practitioner staff, is opposed both to the original objects of the founder and to the needs of the locality.

I am, Sir,
Faithfully yours,
F. R. Humphreys,
Chairman, Hampstead Division,
British Medical Association.

